# Differentiation of Prions from L-type BSE versus Sporadic Creutzfeldt-Jakob Disease

**DOI:** 10.3201/eid1812.120342

**Published:** 2012-12

**Authors:** Simon Nicot, Anna Bencsik, Eric Morignat, Nadine Mestre-Francés, Armand Perret-Liaudet, Thierry Baron

**Affiliations:** Author affiliations: Agence Nationale de Sécurité Sanitaire (Anses), Lyon, France (S. Nicot, A. Bencsik, E. Morignat, T. Baron);; INSERM U710, Montpellier, France; Université Montpellier 2, Montpellier; École Pratique des Hautes Études, Paris, France (N. Mestre-Francés);; Hôpitaux Civils de Lyon, Université Lyon 1, INSERM U1028, and Centre National de la Recherche Scientifique, Lyon (A. Perret-Liaudet)

**Keywords:** prions, Creutzfeldt-Jakob disease, sporadic, sCJD, bovine spongiform encephalopathy, BSE, L-type, strain, bioassay, prions and related diseases, lemur, hamster, human

## Abstract

We compared transmission characteristics for prions from L-type bovine spongiform encephalopathy and MM2-cortical sporadic Creutzfeldt-Jakob disease in the Syrian golden hamster and an ovine prion protein–transgenic mouse line and isolated distinct prion strains. Our findings suggest the absence of a causal relationship between these diseases, but further investigation is warranted.

Among transmissible spongiform encephalopathies (TSEs), the L-type bovine spongiform encephalopathy (L-BSE) in cattle requires particular attention for public health. L-BSE is transmitted more efficiently than is classical BSE among primates (*1*–*3*) as well as among transgenic mice that express human prion protein (PrP) (*4*,*5*). We recently reported that L-BSE was readily transmissible by experimental oral inoculation in a nonhuman primate species, the grey mouse lemur (*Microcebus murinus*) (*3*). These findings raise the possibility that some human Creutzfeldt-Jakob disease (CJD) cases might result from exposure to the L-BSE agent; previous studies highlighted similarities between L-BSE and some human subtypes (type 2) of sporadic CJD (sCJD) (*1*,*6*).

To examine the possible relationship between L-BSE and sCJD, we evaluated a strain-typing strategy that relies on comparative transmission characteristics in the Syrian golden hamster and in a transgenic mouse line (TgOvPrP4) expressing ovine PrP (ARQ allele). Both of these species are susceptible to L-BSE prions from cattle (*7*,*8*). The transmission of L-BSE, including after a first passage in *Microcebus murinus* lemurs (*3*), was compared with that for the MM2-cortical subtype of sCJD (*9*); this subtype was chosen on the basis of a study that indicated higher levels of molecular similarities of L-BSE with this sCJD subtype than with the MV2 subtype (*1*).

## The Study

The TSE brain inocula used in this study, conducted during November 2010–December 2011, were derived from 2 natural L-BSE isolates from France (02-2528 and 08-0074); a lemur injected intracerebrally (i.c.) with the 02-2528 L-BSE cattle isolate (*3*); and a human patient with MM2-cortical sCJD. Consent was obtained for using tissues from the human patient in research, including genetic analyses. Animal experiments were performed in the biohazard prevention area (A3) of the Anses-Lyon animal facilities, in accordance with the guidelines of the French Ethical Committee (decree 87-848) and European Community Directive 86/609/EEC. 

Six-week-old TgOvPrP4 mice and 4-week-old Syrian golden hamsters were injected i.c. with 20 and 30 µL, respectively, of 10% (wt/vol) brain homogenates in 5% sterile glucose. Serial passages were performed in TgOvPrP4 mice by i.c. inoculation of 1% (wt/vol) homogenates from mice positive for protease-resistant PrP (PrP^res^). At the terminal stage of the disease, animals were euthanized, and their brains and spleens were collected for PrP^res^ analyses by Western blot and for histopathologic studies (*8*). 

**Figure 1:**
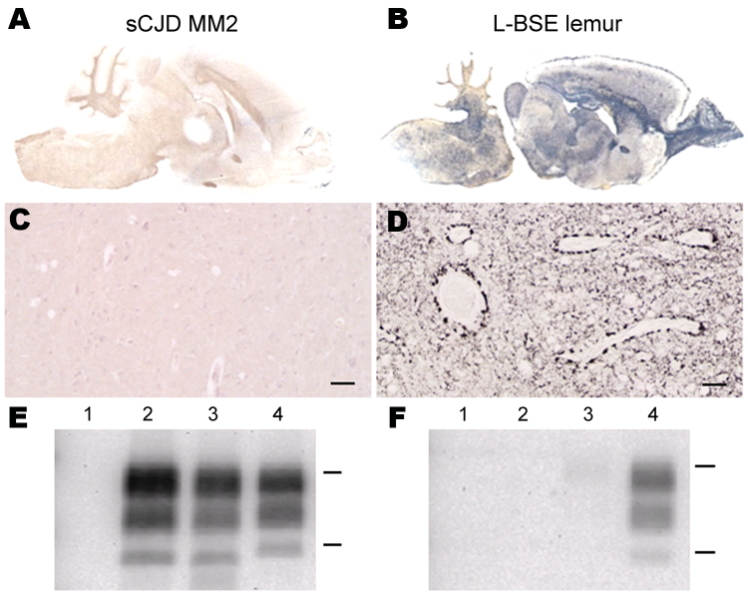
Susceptibility of Syrian golden hamsters to MM2-cortical subtype sporadic Creutzfeldt-Jakob disease (sCJD) and L-type bovine spongiform encephalopathy (L-BSE) prions. Disease-associated prion protein (PrP^d^) was analyzed in brains of hamsters injected with human MM2-cortical sCJD and L-BSE from a mouse lemur by paraffin-embedded tissue blot (A, B), immunohistochemistry (C, D), or Western blot (E, F). Monoclonal antibodies against prion protein were SAF84 (A–D), SHa31 (E), and 12B2 (F). C, D) Scale bars = 200 µm. E, F) Controls were hamsters infected with L-BSE from cattle (isolate 02-2528) and with scrapie (experimental isolate SSBP/1 after a first passage in ovine prion protein–transgenic mice). Lane 1, sCJD MM2; lane 2, L-BSE from lemur; lane 3, L-BSE from cattle control; lane 4, scrapie control. Bars to the right indicate the 29.0- and 20.1-kDa marker positions.

In hamsters, transmission of the MM2-cortical sCJD agent was inefficient. Clinical signs were absent up to 876 days postinoculation (dpi) (Table), and disease-associated PrP (PrP^d^) in brain samples was not detected by paraffin-embedded tissue blot (PET-blot) (Figure 1, panel A), immunohistochemical (Figure 1, panel C), or Western blot (Figure 1, panels E, F) analyses. PrP^res^ was also undetectable in spleen tissues by Western blot (Table). 

**Table:** Comparison of transmission of sCJD and L-BSE in hamsters and mice

Hosts and inoculum	Passage	Mean survival time, dpi ± SD	No. brain PrP^d^ positive/no. tested	No. spleen PrP^res^ positive/no. tested
Syrian golden hamsters				
sCJD MM2-cortical	1	833 ± 33	0/4	0/4
L-BSE lemur	1	529 ± 177	5/5	1/4
L-BSE cattle (02-2528)	1	622 ± 64†	4/5†	0/5
TgOvPrP4 mice				
sCJD MM2-cortical	1	639 ± 49	3/4	0/4
L-BSE lemur	1	509 ± 97	7/7	7/7
L-BSE cattle (02-2528)	1	627 ± 74‡	9/10‡	0/5§
L-BSE cattle (08-0074)	1	497 ± 49	6/8	0/9
sCJD MM2-cortical	2	111 ± 25	12/12	12/12
L-BSE lemur	2	194 ± 7	12/12	12/12
L-BSE cattle (02-2528)	2	202 ± 26‡	9/9‡	3/5
L-BSE cattle (08-0074)	2	186 ± 37	12/12	9/11

*Isolate identification numbers are shown in parentheses. sCJD, sporadic Creutzfeldt-Jakob disease; L-BSE, L-type bovine spongiform encephalopathy; TgOvPrP4, ovine prion protein–transgenic; dpi, days postinoculation; PrP^d^, disease-associated prion protein; PrP^res^, protease-resistant prion protein. †Data from (7). ‡Data from (8). §Data from (10).

In contrast, the L-BSE agent passaged in a lemur was efficiently transmitted to hamsters, with a mean survival period of 529 ± 117 dpi, similar to that for L-BSE from cattle (622 ± 64 dpi) (Table). PET-blot analysis (Figure 1, panel B) showed widespread PrP^res^ distribution in the brain; immunohistochemical analysis (Figure 1, panel D) showed a granular type of PrP^d^ deposition that redefined the periphery of most of the blood vessels. Western blot analysis (Figure 1, panels E, F) showed PrP^res^ in the brains of hamsters inoculated with L-BSE from cattle and lemur and in 1/4 spleens of hamsters injected with L-BSE passaged in lemur (Table). Brain PrP^res^ was characterized by low apparent molecular mass (≈19 kDa for the unglycosylated band) associated with a lack of reactivity toward the N terminal 12B2 antibody, in contrast to that for the control animal with scrapie (Figure 1, panels E, F).

In TgOvPrP4 mice, all TSEs were efficiently transmitted, as confirmed by PrP^d^ accumulation in the mouse brains (Table). After serial passages in additional TgOvPrP4 mice, the survival periods in each experiment became considerably shorter (Table; Technical Appendix Figure 1). No statistically significant differences in results were identified between the L-BSE sources (p>0.6). Mean survival period decreased to 111 ± 25 dpi at second passage in mice inoculated with the agent of MM2-cortical subtype sCJD, which differed significantly from that of mice inoculated with L-BSE (p<0.0001). A third passage of both cattle L-BSE and human sCJD did not reduce the survival periods in TgOvPrP4 mice (data not shown).

**Figure 2:**
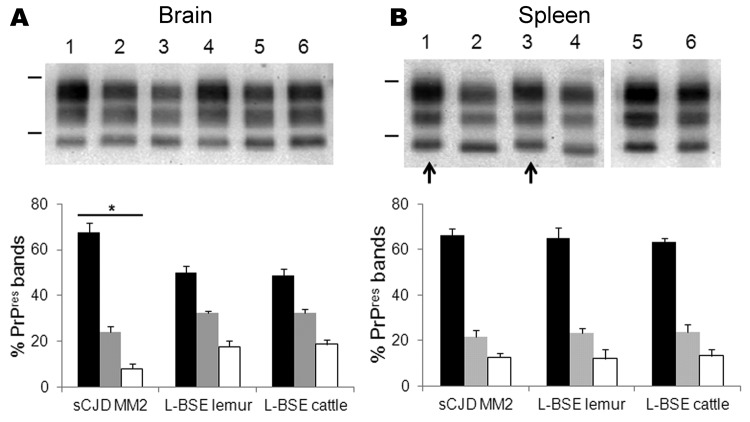
Western blot molecular typing of protease-resistant prion protein (PrP^res^) in brain and spleen tissues of ovine prion protein–transgenic (TgOvPrP4) mice at second passage. PrP^res^ from mice infected with MM2-cortical subtype sporadic Creutzfeldt-Jakob disease (sCJD), L-type bovine spongiform encephalopathy (L-BSE) from lemur, and L-BSE from cattle (02-2528) were compared in brain (A) and spleen (B) tissues (monoclonal antibody SHa31). Bars to the left of Western blots indicate the 29.0- and 20.1-kDa marker positions. A) Lanes 1, 4, sCJD MM2; lanes 2, 5, L-BSE from lemur; lanes 3, 6, L-BSE from cattle control; B) lanes 1, 3, sCJD MM2; lanes 2, 4, 6, L-BSE from lemur; lane 5, L-BSE from cattle control. C, D) Proportions of PrP^res^ glycoforms in brain (C) and spleen (D) tissues. Error bars indicate SD. *Indicates p<0.0001 when comparing PrP^res^ proportions from mice infected with MM2-cortical sCJD with those infected with L-BSE.

Western blot analyses of PrP^res^ from mouse brains showed partially similar features for MM2-cortical sCJD and L-BSE, including low molecular mass (≈19 kDa for the unglycosylated band) (Figure 2, panel A) and similar conformational stability of PrP^d^ after treatment with guanidinium hydrochloride (Technical Appendix Figure 2). However, the proportions of diglycosylated, monoglycosylated, and unglycosylated bands of brain PrP^res^ differed between sCJD and L-BSE (Figure 2, panel C); higher proportions of diglycosylated PrP^res^ were found in sCJD-infected mice (mean 67% of the total signal) compared with L-BSE–infected mice (≈18% lower; p<0.0001). PrP^res^ was readily identified in the spleens of TgOvPrP4 mice at the second passage for sCJD and L-BSE from cattle and at the first passage for L-BSE from lemur (Table). No significant differences in the proportions of PrP^res^ glycoforms for sCJD-infected versus L-BSE–infected mice were observed in the spleens (Figure 2, panel D), but PrP^res^ was ≈0.5 kD higher in mice injected with sCJD (Figure 2, panel B, arrows).

Histopathologic analysis showed severe vacuolar lesions in TgOvPrP4 mice infected at second passage with sCJD and lemur-passaged L-BSE (Technical Appendix Figure 3). However, in sCJD-infected mice, vacuolar lesions were mostly observed in the anterior parts of the brain (except the parietal cortex), whereas in mice infected with lemur-passaged L-BSE, the lesions were more widely distributed, involving the colliculi and the hypothalamus. In mice infected with sCJD and lemur-passaged L-BSE, PET-blot analyses showed that most of the PrP^res^ occurred in the frontal parts of the brain, but the intensity and appearance of PrP^res^ in the cortex, thalamus, and hippocampus were distinctly different. Immunohistochemical analyses of the hippocampus showed PrP^d^ deposition in the dentate gyrus in sCJD-infected mice, in contrast to a lack of deposition in lemur-passaged L-BSE–infected mice.

## Conclusions

We report the isolation of 2 prion strains derived from L-BSE and MM2-cortical sCJD after transmission in Syrian hamsters and ovine PrP–transgenic mice. In hamsters, we did not transmit any disease with sCJD, but the transmission of L-BSE from lemur was efficient, as previously reported for L-BSE from cattle (*7*,*11*). This result suggests that L-BSE did not undergo major modifications after this cross-species transmission and could indicate a clear biologic difference between MM2-cortical sCJD and L-BSE. We also demonstrated the efficient transmission of both L-BSE and MM2-cortical sCJD in TgOvPrP4 mice, which enabled us to compare these diseases in a single model. Unexpectedly, during serial passages, we observed that the agent of MM2-cortical sCJD causes a much more rapidly fatal disease. Despite similar molecular features in sCJD and L-BSE, including the PrP^res^ electrophoretic mobility and the conformational stability of PrP^d^, sCJD and L-BSE differed in PrP^res^ glycosylation for the mouse brains and gel migrations for the mouse spleens. Mice infected with MM2-cortical sCJD versus those infected with L-BSE also showed distinct lesion profiles and PrP^d^ distribution, which confirms clear biologic differences between these diseases.

Although only 1 case of sCJD of a unique molecular subtype was examined in our study, our observations do not support the hypothesis of a causal relationship between L-BSE and this human sCJD subtype. Our study thus encourages further investigations using the proposed bioassay approach for a more complete evaluation of possible relationships between L-BSE and human prion diseases.

Technical Appendix Kaplan Meier survival curves for mice injected with MM2-cortical sporadic Creutzfeldt-Jakob disease (sCJD), L-type bovine spongiform encephalopathy (L-BSE) from lemur, L-BSE from cattle, and classical BSE; conformational stability assay of disease-associated prion protein in brains of TgOvPrP4 mice at second passage; and histopathological features of MM2-cortical sporadic sCJD and L-BSE from lemur transmitted to ovine prion protein–transgenic mice at second passage.
