# Supplementary material for: Differentiation of Prions from L-type BSE versus Sporadic Creutzfeldt-Jakob Disease
Source: Emerg Infect Dis. 2012 Dec;18(12):2028–31. doi: 10.3201/eid1812.120342 (PMC3557863; doi:10.3201/eid1812.120342)
Supplement: Technical Appendix — Kaplan Meier survival curves for mice injected with MM2-cortical sporadic Creutzfeldt-Jakob disease (sCJD), L-type bovine spongiform encephalopathy (L-BSE) from lemur, L-BSE from cattle, and classical BSE; conformational stability assay of disease-associated prion protein in brains of TgOvPrP4 mice at second passage; and histopathological features of MM2-cortical sporadic sCJD and L-BSE from lemur transmitted to ovine prion protein–transgenic mice at second passage. [file 12-0342-Techapp-s1.pdf]

Article DOI: http://dx.doi.org/10.3201/eid1812.120342

Differentiation of Prions from L-type BSE Versus Sporadic Creutzfeldt-Jakob Disease

Technical Appendix

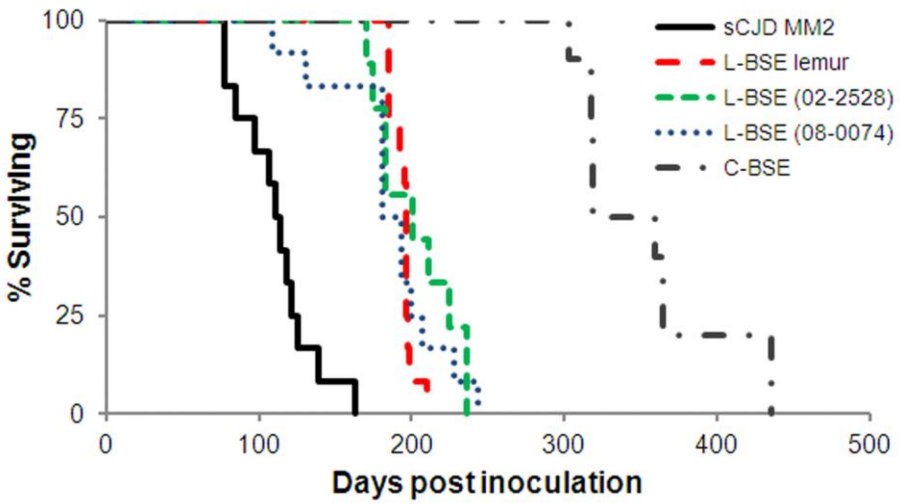

Technical Appendix Figure 1. Kaplan Meier survival curves for mice injected with MM2-cortical sporadic Creutzfeldt-Jakob disease (sCJD), L-type bovine spongiform encephalopathy (L-BSE) from lemur, L-BSE from cattle (02-2528 and 08-0074 isolates), and classical BSE (01-2281 isolate).

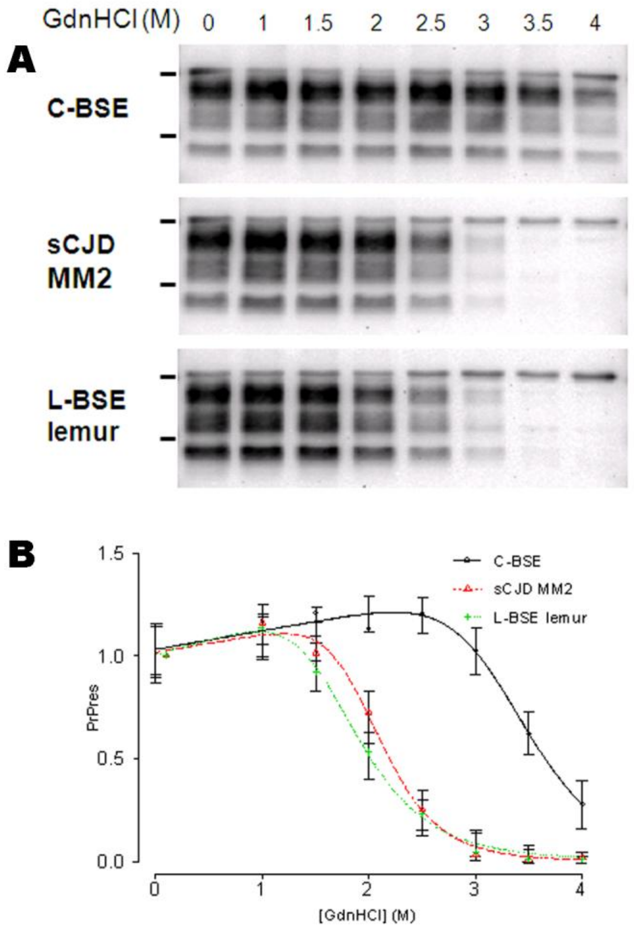

Technical Appendix Figure 2. Conformational stability assay of disease-associated prion protein (PrP^{d}) in brains of TgOvPrP4 mice at second passage. PrP^{d} was denatured with increasing concentrations (0 to 4 M) of guanidinium hydrochloride (GdnHCl) followed by digestion with proteinase K. Prion sources were classical bovine spongiform encephalopathy (C-BSE, 01-2281 isolate), MM2-cortical sporadic Creutzfeldt-Jakob disease (sCJD), and L-type BSE from lemur. GdnHCl denaturation curves were plotted as protease-resistant prion protein (PrP^{res}) Western blot signals (monoclonal antibody SHa31) (A) and fitted to a 5-parameter Brain-Cousens modified log-logistic model (B). The same tissue equivalents (0.4 mg) were loaded in each lane. Bars to the left of Western blots indicate the 29.0- and 20.1-kDa marker positions.

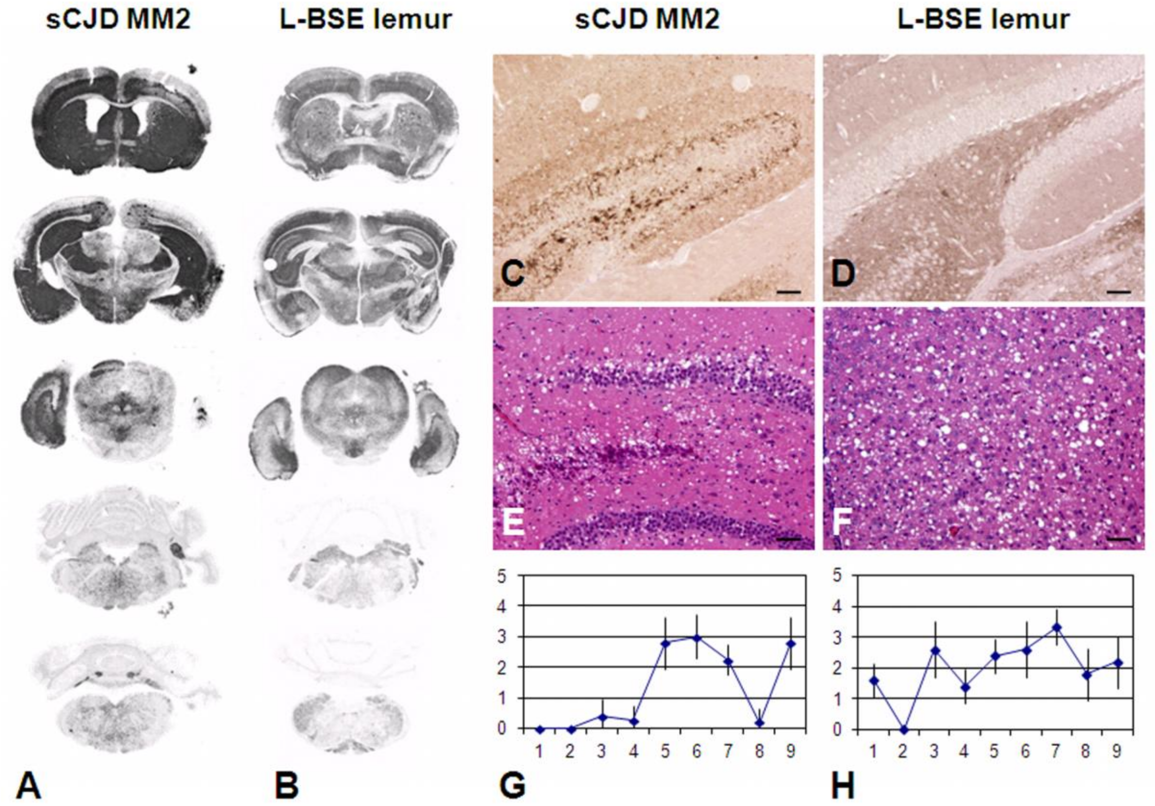

Technical Appendix Figure 3. Histopathological features of MM2-cortical sporadic Creutzfeldt-Jakob disease (sCJD) and L-type bovine spongiform encephalopathy (L-BSE) from lemur transmitted to ovine prion protein–transgenic mice at second passage. A, B) Paraffin-embedded tissue blot analysis (monoclonal antibody SAF84) of coronal brain sections. C, D) Immunohistochemical detection (monoclonal antibody SAF84) of disease-associated prion protein (PrP^d) in the hippocampus (dentate gyrus). Scale bars = 200 µm. E, F) Vacuolar lesions after hematoxylin-eosin staining in the hippocampus and thalamus, respectively. Scale bars = 200 µm.G, H) Lesion profiles in mice infected with sCJD MM2 (n = 5) and L-BSE from lemur (n = 6) respectively. 1. dorsal medulla nuclei, 2. cerebellar cortex, 3. superior colliculus, 4. hypothalamus, 5. central thalamus, 6. hippocampus, 7. septal nuclei, 8. cerebral cortex at the level of the thalamus (parietal cortex), 9. cerebral cortex at the level of the septal nuclei (frontal cortex).
